# Death of Dr. Brigham

**Published:** 1849-11

**Authors:** 


*Death of Dr. Brigham.*—Death has been busy, of late, in the ranks of the medical profession. In the list of several distinguished members lately deceased, we have now to add the name of Dr. Amariah Brigham, Superintendent of the Lunatic Asylum, in Utica, N. Y. Dr. Brigham, early in his professional career, acquired an extensive reputation as the author of several works on subjects connected with medicine, hygiene, and education. During the latter period of his life, his labors were directed exclusively to insanity, and with this department of medical science, his name has become identified. He leaves, in his name and character, a more valuable legacy than riches.

We learn that the duties of Superintendent of the Lunatic Asylum, at Utica, are, at present, performed by Prof. Charles B. Coventry.

Prof. Coventry has devoted much attention to the subject of insanity, and has been connected with the Utica Asylum, as one of the trustees, ever since its existence. The Institution has been truly fortunate in securing his valuable services until a permanent appointment is made.

